# Flourishing and Functional Difficulties among Autistic Youth: A Confirmatory Factor Analysis

**DOI:** 10.3390/children11030325

**Published:** 2024-03-09

**Authors:** Lauren M. Little, Laura-Lee Schwefel

**Affiliations:** 1 Department of Occupational Therapy, College of Health Sciences, Rush University, Chicago, IL 60612, USA; 2 Department of Health Sciences, College of Health Sciences, Rush University, Chicago, IL 60612, USA; laura_l_schwefel@rush.edu

**Keywords:** autism, flourishing, functional difficulties

## Abstract

The International Classification of Functioning, Disability, and Health for Children and Youth outlines body structures and functions and activities and participation to fully describe elements that support or detract from participation. While flourishing has gained attention in recent literature, research also points to the role of functional difficulties among autistic youth in influencing participation. Clearly, function is a multi-dimensional and complex construct and likely consists of both indicators of flourishing and functional difficulties. We used data from the National Survey of Children’s Health (NSCH) from 2016 to 2020 to identify aspects of flourishing functional difficulties to achieve the following aims: (1) Investigate the factor structure of flourishing and functional difficulties among autistic youth ages 10–17 years; and (2) examine the extent to which child variables (i.e., sex, age, race, ethnicity, autism severity, poverty) are associated with flourishing and functional difficulties. Autistic children (n = 2960) between the ages of 10 and 17 years were included. We used confirmatory factor analysis followed by a multivariate general linear model (GLM) to examine the association between child variables and factors. Results indicated a six-factor structure (medical conditions, instrumental activities of daily living, activities of daily living, social competence, behavioral control, and school motivation) with good model fit (root mean square error of approximation = 0.08 [*p* = 0.926], comparative fit index = 0.94, Tucker–Lewis index = 0.91). Multivariate GLM showed that child factors were differentially and significantly associated with factors of functional difficulties and flourishing. Current findings suggest that 16 items measured by the NSCH result in a six-factor structure of flourishing and functional difficulties among autistic youth. A comprehensive approach to capture function among autistic youth must assess aspects of flourishing and difficulties.

## 1. Introduction

Autistic youth experience differences in social communication and interaction and demonstrate restricted interests. Estimates report the rising prevalence of autism diagnoses is 1 in 36 children [1], and diagnostic criteria for autism include a continuum of three levels of support (i.e., requiring very substantial support, requiring substantial support, requiring support) [2]. Due to the varying levels and types of necessary supports, the heterogeneity of autism complicates tailored and personalized interventions. For example, traditional measures of cognitive function (e.g., intelligence quotient [IQ]) do not consistently correlate with adaptive behavior and function among autistic youth [3,4,5,6]. Additionally, there is great variability in communication among autistic youth (pre-verbal, augmentative and alternative communication, verbal) which likely influences opportunities for participation in everyday activities. Therefore, research points to the importance of the identification of patterns of function among individuals with autism to identify how child flourishing and functional difficulties contribute to everyday participation.

The International Classification of Functioning, Disability, and Health for Children and Youth (ICF-CY) [7] outlines body structures and functions and activities and participation to fully describe elements that support or detract from participation. The ICF-CY is a strengths-based approach placing function at its core. The ICF-CY highlights the dynamic interactions between a person’s health and their functional abilities which influence participation in life experiences. Contextual factors (environments, families) influence child factors, activities, and participation and may be considered facilitators or barriers to engagement in life experiences. In this study, we used the ICF-CY to identify specific behaviors, classified as flourishing and functional difficulties, to ultimately describe how the combination may contribute to overall function of autistic youth.

Flourishing, also referred to as functional strengths or thriving, is multifaced and encompasses a myriad of dimensions leading to positive health outcomes characteristically expressed as competence, self-regulation, self-worth, and curiosity [8,9]. When children show curiosity in learning, ability to self-regulate emotions, and persistence in goal achievement, such skills foster positive experiences by way of meaning and engagement [10]. Additionally, flourishing is inversely related adverse child circumstances such as social vulnerability [10]. Flourishing arises out of an intrinsic motivation to pursue a sense of purpose and meaning in one’s environment, and supports co-regulation through positive relationships, engagement through interest and curiosity, and reciprocal communication through practice expressing wants, needs, and preferences both verbally and non-verbally. Protective (e.g., family support and caregiver responsiveness, stimulating environment, safe neighborhood, religious involvement) and risk factors (e.g., domestic violence, family stress, parental unemployment, negative peers, inadequate recreational activities) also influence flourishing markers and help positively or negatively define well-being in physical health and functioning, mental and emotional homeostasis, and cognitive, academic, and social development [9].

Flourishing in children without developmental conditions is an optimal marker for promoting overall health and well-being reinforced through associations to family resilience and neighborhoods. However, only one in four children without developmental conditions met flourishing criteria from the 2016–2017 National Survey of Children’s Health (NSCH) yet strong associations were established between family resilience and childhood flourishing [10]. Contextual factors as they interact with family and child factors, may influence child flourishing, and findings point to the importance of protective factors, such as family resilience, in facilitating nurturing relationships, and environments for child well-being [11,12]. For example, in another study on children without developmental conditions, flourishing during the COVID-19 pandemic in 2020 resulted in a 12% decrease in flourishing, likely due to disruptions in learning, routines, play, school activities, and social engagement [13]. 

Research on flourishing among autistic youth remains limited. Hilton et al. (2019) conducted a confirmatory factor analysis (CFA) using the 2016–2017 NSCH, among youth with and without autism. Results identified a three-factor construct of flourishing: social competence (i.e., bullied, bullied others, argues too much, makes friends), Behavioral Regulation (i.e., curiosity, finishes tasks, stays calm and shares ideas), and school motivation (i.e., homework, does well in school). Researchers compared differences in flourishing between youth with and without autism and found that autistic youth reported lower scores in social competence and behavioral regulation for autistic children [14]. However, indicators of flourishing may differ between children with and without autism. Ross et al. (2023) argued that this fundamental difference may introduce measurement bias into interpretations that compare autistic youth to those with typical development. Authors caution that when inferring autistic children’s flourishing patterns is complex since parent perceptions of flourishing may not be a true indication of an autistic child’s experiences relative to their developmental trajectory. Additionally, interpretations comparing autistic children’s flourishing experiences with neurotypical children overlooks the diversity of expression inherent in a spectrum condition failing to consider the importance of a strengths-based frame of reference [15]. Overall, identifying indicators of flourishing is crucial to understand strengths of autistic children, their families, and their communities. Exploration of constructs related to relationships. internal and external motivation (e.g., drive, persistence, focus), self-regulation, and task completion may present a more holistic view of child function to inform best practice models for promoting strengths among autistic individuals.

In the context of health, functional difficulties may negatively influence participation in society and life experiences. Functional difficulties are described differently across the literature. Some studies consider social participation [16] and remembering/concentrating, communicating, and self-care as functional difficulties [17]. Other studies consider vision, hearing, self-care, use of hands, dressing, learning, communication, running errands alone, and/or behavior problems as falling within functional difficulties [18,19]. Within the ICF-CY, there is a difference between categories of (1) body structures and functions and (2) activity and participation. The range of functional difficulties falls within both categories; that is, some functional difficulties are likely related to medical conditions (e.g., difficulty breathing) while other difficulties arise during participation in everyday activities (e.g., difficulty running errands alone).

Studies have outlined how functional difficulties influence participation among autistic youth. One study identified a positive relationship between motor challenges and daily living activities, which affected child dressing, bathing, home and school self-management, and meal preparation [20]. Additionally, other studies have shown how mastery of daily living skills such as management of one’s own health (e.g., brushing teeth, medication management) and home (e.g., cleaning, cooking) including community engagement (e.g., finding and maintaining a job) are required for independent living but are often impaired in individuals with ASD [21,22]. Motor challenges are under-recognized in ASD children and although not a diagnostic criterion, praxis and motor coordination discrepancies commonly identified in ASD children confound functional activities such as self-care, leisure, social communication, and learning [23,24,25]. For a holistic view of function and participation among autistic youth, both flourishing and functional difficulties must be considered. A greater understanding of ASD functional profiles and variations is central to maximizing intervention strategies that promote consider the impact of functional limitations or restrictions on everyday life experiences and engagement.

By definition, heterogeneity in autism contributes to differences in child function and participation; however, it is unclear how child and family factors influence differences in child flourishing and functional difficulties. Indicators of flourishing may overlap with diagnostic characteristics of ASD [15]. For example, if flourishing is partially considered as social participation, the social communication difficulties in autism predispose children to perform ‘worse’ on indicators of flourishing Therefore, authors have argued that typical measures to assess flourishing may not be the most appropriate ascertain similar dimensions for autistic children [26]. To establish appropriate measures of autistic flourishing, it may be necessary to understand variability in flourishing by child and family characteristics. For example, one study found that younger children engaged in certain behaviors consistent with flourishing less frequently than older autistic children [26]. Additionally, sex differences in autism contribute to heterogeneity with some studies suggesting that autistic females present with fewer social differences [25] and more motor concerns [27]. Last, research shows that autistic children from under-resourced backgrounds and communities may lack access to support services [28], which negatively influences developmental trajectories and may contribute to limited flourishing and increased functional difficulties. Clearly, an understanding of how child and family factors influences specific elements of flourishing and functional difficulties among autistic youth is needed.

Given the importance and multi-dimensional nature of function among autistic youth, the purpose of this study was to investigate the factor structure of variables associated with flourishing and functional difficulties, which may load on to a common construct of child function. Specifically, we used a confirmatory factor analysis (CFA) to test 10 indicators of flourishing and 7 indicators of functional difficulty. Additionally, we tested the extent to which child characteristics (i.e., sex, age, race, ethnicity, autism severity, poverty) were associated with flourishing and functional difficulties factors.

## 2. Materials and Methods

### 2.1. Participants

Participant data was derived from the NSCH, a large population-based survey administered to households nationwide. The purpose of this annual survey is to gather information describing the health experiences of all children in the United States. Data from children aged 10–17 years from combined survey years 2016–2017, 2018–2019, and a single year 2020 were used in the current study. The NSCH is completed by a household member (62% mother, 30% father, 8% other guardian) who is most familiar with their child’s health and health care needs [29]. Inclusion criteria consisted of children aged 10–17 years with a diagnosis of autism spectrum disorder. Children with comorbid blindness and/or deafness were excluded. See Table 1. 

### 2.2. Data Analysis

#### 2.2.1. Data Integration and Transformation

Given the differences in item level response values between NSCH years 2016–2017 and 2018–2020, we used several steps to integrate and transform data. First, we downloaded each data file. After pulling each variable of interest from each cohort’s data file, we consulted NSCH codebooks to ensure that transformation across cohorts was accurate and meaningful.

Calculations for flourishing have varied by NSCH year(s) and analyses [14,15]. In 2016–2017 and 2018–2019, NSCH considered the flourishing construct based on three items: (1) show interest and curiosity in learning new things, (2) work to finish tasks he or she starts, and (3) stay calm and in control when faced with a challenge. However, 3 response categories in 2016–2017 (always/almost always, sometimes, never) were parsed into 4 response categories in 2018–2020 (always, usually, sometimes, never). Therefore, all flourishing items from 2018 to 2020 were combined into 3 response categories to match those from 2016 to 2017 and create consistency across all datasets. Based on the results of Hilton et al. (2019) [14], we investigated 10 items as they fell into the flourishing construct: (1) Is bullied, picked on, or excluded by other children; (2) Bullies others, picks on them or excludes them: (3) Has difficulty making and keeping friends; (4) Shows interest and curiosity in learning new things; (5) Works to finish tasks he or she starts; (6) Stays calm and in control when faced with a challenge; (7) How well can you and this child share ideas or talk about things that really matter; (8) Does all required homework; (9) Cares about doing well in school; and (10) Argues too much.

Functional difficulties did not differ by cohort and were therefore integrated as a binary variable (i.e., yes/no) across 2016–2020. According to the NSCH, functional difficulty items include 9 body structures and functions that span 6–17 years. We chose to examine the following: (1) Frequent or chronic difficulty with breathing or other respiratory problems; (2) Eating or swallowing; digesting food, including stomach/intestinal problems, constipation, or diarrhea; (3) Repeated or chronic physical pain, including headaches or other back or body pain; (4) Serious difficulty concentrating, remembering, or making decisions; (5) Serious difficulty walking or climbing stairs; (6) Difficulty dressing or bathing; and (7) Difficulty doing errands alone, such as visiting a doctor’s office or shopping. We did not examine functional difficulty items related to blindness or deafness because children with comorbid blindness and/or deafness were excluded.

For a measure of sex and age, the NSCH identifies each child as male or female and provides age in years. For race and ethnicity, the NSCH provided 5 categories, Asian, Black/African American, White, Hispanic, or Multi-racial/Other. Autism severity is categorized into ‘mild’ or ‘moderate-severe’. Federal poverty level is calculated according to the participant’s State Health Insurance Program.

#### 2.2.2. Confirmatory Factor Analysis

To address Aim 1 (i.e., Investigate the factor structure of flourishing and functional difficulties among autistic youth ages 10–17 years) we used a CFA approach in Mplus 8.4 [30] to estimate all models. Factor analysis is used to assess the relationships between item-level data and underlying latent variables (factors). In factor analysis, item level data is viewed as representative of underlying latent variables. In the current investigation, the unobserved latent variables are representative of functional difficulties and flourishing. We used CFA with weighted least squares with adjustments for the mean and variance (WLSMV) estimation. All items were allowed to correlate within factor. In the dataset, there were 23 missing values which were not imputed and excluded from analyses. The factor structure(s) was/were confirmed by CFA using model fit indices, including the comparative fit index (CFI < 0.95 for good fit), the Tucker–Lewis index (TLI < 0.95 for good fit), the root mean square error of approximation (RMSEA < 0.08 for adequate fit), and the standardized root mean square residual (SRMR < 0.08 for good fit) as well as factor loadings (λ > 0.4 for acceptable factor loadings) [31]. We did not perform a power analysis for the current CFA; however, we had a relatively large sample size, which is suggested for a CFA (e.g., over n = 200). [32] Moreover, the RMSEA value has been shown relatively insensitive to sample size [33].

#### 2.2.3. The Multivariate General Linear Model

To address Aim 2 (i.e., examine the extent to which child variables are associated with flourishing and functional difficulties), we used a multivariate general linear model. We tested the main effect of child characteristics (sex, age, race/ethnicity, autism severity, poverty) as independent variables on the dependent factors associated with flourishing and functional difficulty factor scores derived from Aim 1. We used Bonferroni follow up comparisons.

## 3. Results

### 3.1. Confirmatory Factor Analysis

Model results initially showed poor model fit with the inclusion of the ‘argues too much’ item, which would not load cleanly on social competence as previous investigations suggest [14,15]. Instead, the item showed a low loading value across multiple factors and weas therefore removed from the final model. Additionally, ‘make friends’ was allowed to load on IADLs and social competence for better model fit. Results indicated a six-factor structure (medical, IADLs, ADLs, social competence, behavioral control, and school motivation) with good model fit. See Table 2.

All factor loadings ranged from 0.560 to 0.974 (see Table 3) and correlations between factors were −0.701 to 0.835 (see Table 4). Specifically, factors associated with functional difficulties (medical difficulties, IADLs, ADLs) were largely positively associated with each other and negatively associated with flourishing (social competence, behavioral control, school motivation). Overall, these associations demonstrate that flourishing is negatively associated with functional difficulties among autistic youth.

### 3.2. Multivariate GLM

Multivariate GLM results showed significant main effects on sex (Pillai’s Trace = 0.011, F [6,6] = 5.298, *p* < 0.001), race/ethnicity (Pillai’s Trace = 0.032, F [6,24] = 3.953, *p* < 0.001), autism severity (Pillai’s Trace = 0.225, F [6,6] = 141.857, *p* < 0.001), poverty (Pillai’s Trace = 0.018, F [6,18] = 2.995, *p* < 0.001, and a non-significant effect of age (Pillai’s Trace = 0.004, F [6,6] = 1.886, *p* = 0.080). With regard to specific factors, see Table 5.

### 3.3. Follow up Comparisons

**Figure 1:**
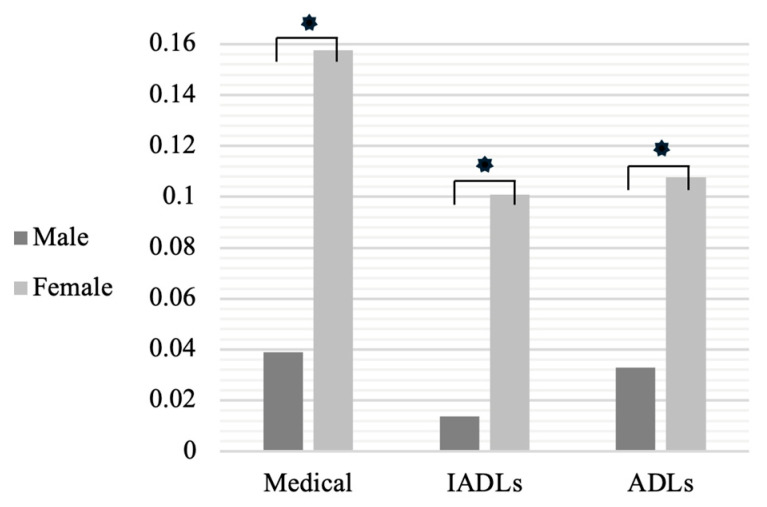
Functional Difficulty Differences by Sex. * *p* < 0.05.

Males differed from females in medical difficulties, IADLs, and ADLs (all *p* < 0.01, respectively). See Figure 1.

Children rated as ‘mild’ as compared to ‘moderate-severe’ significantly differed on all factors, including medical, IADLs, ADLs, Social, Behavior, School (all *p <* 0.001). Those in the moderate-severe group showed significantly more functional difficulties and decreased flourishing.

**Figure 2:**
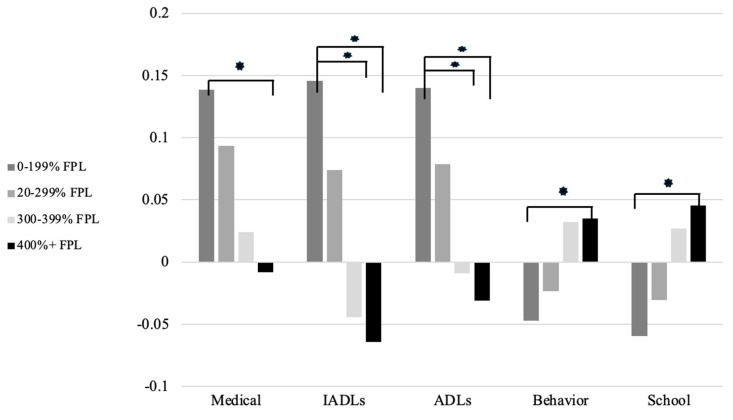
Flourishing and functional difficulty factor scores by poverty level. * *p* < 0.05. Note: Higher factor scores on medical, IADLs, and ADLs indicate increased difficulties while increased factor scores on Behavior and School indicate increased flourishing.

In medical difficulties, children in 0–199% of poverty significantly differed from those with 400% or greater (*p* < 0.05). In IADLs and ADLs, children in 0–199% of poverty differed from those with 300–399% and 400% or greater (all *p* < 0.05, respectively). Children in 0–199% of poverty significantly differed from those with 400% or greater in behavior (*p* < 0.01) and school (*p* < 0.05). Poverty was not significantly associated with social competence. See Figure 2.

With regard to race/ethnicity, Black children were significantly different from all other races except Asian on medical complications (all *p* < 0.05, respectively), with black children showing decreased medical functional difficulties than other racial groups. Black children also differed from White and Multi-racial/other children on social competence (*p* < 0.05), showing better social competence. Races did not significantly differ on ADLs, IADLS, behavior, or school.

## 4. Discussion

Novel findings from the current study illuminate flourishing behaviors and functional difficulties that describe a common construct of function among autistic youth. Specifically, flourishing (social competence, behavioral control, and school motivation) and functional difficulties (medical, IADLs, ADLs) work together to provide an estimate of overall function. Factors associated with flourishing and functional difficulties were inversely associated, which means that when children have fewer functional difficulties, they are more likely to demonstrate behaviors consistent with flourishing. Additionally, current findings point to the differential influence of child sex, autism severity level, federal poverty level, and race/ethnicity on child function.

Regarding flourishing, current findings align with previous investigations however, ‘argues too much’ did not align with social competence and was therefore not included in the final model. Additionally, item loadings on the flourishing factors were similar to the findings of Hilton et al. (2019) [14], although loadings in the current analysis were slightly increased. This may be due to the focus on the autistic group only (i.e., no inclusion of participants without autism), and may be to the overall investigation and inclusion of functional difficulty items into the analysis as well. That is, when child function is examined from both body structures and functions as well as activities and participation, items associated with flourishing provide a more holistic view [14].

Three factors (medical difficulties, IADLs, ADLs) encompassed functional difficulty items. Categorizing previously disparate functional difficulty items into factors can better inform child function and participation. When functional difficulties are categorized this way, researchers and clinicians may better understand how to design interventions based on the interplay between body functions (medical difficulties) and participation difficulties (IADLs, ADLs). In other words, individual child scores on each factor can help illuminate how chronic pain, for example, influences child ability for dressing and bathing. Through operationalizing functional difficulties, the six factors associated with flourishing and functional difficulties may better translate to real-world applications.

Associations between factors may also inform applications for intervention and research. For example, higher scores in medical difficulties, which encompasses gastrointestinal (GI) symptoms, were associated with lower in social competence and behavioral control. Previous research shows that GI symptoms influence behavior problems in autistic youth [34,35], and GI symptoms may negatively influence social function [36]. Moreover, evidence suggests that when autistic individuals show externalizing behavior, they are less likely to be independent with daily living skills [37,38]. Negative associations between factors of IADLs and behavioral control support such findings, as engagement and completion of independent tasks such as running errands requires self-regulation strategies such as staying clam in the face of challenges.

Findings from the study illuminate how sex differences in autism are related to functional difficulties, specifically medical, ADLs, and IADLs. In the current sample, females showed worse functional difficulties as compared to males. Sex differences in autism are well established, yet etiologies remain elusive [39]. Females with autism have been found to have fewer externalizing and social behavior difficulties [40]; however, decreased social interaction difficulties have been linked to a higher incidence of camouflaging in females [41]. Some research suggests that females with autism have more early motor concerns [27] and fewer behavior and social concerns than males [42]. Additionally, females with autism show worse adaptive function than males even with decreased autism symptoms [43]. Worse scores in medical difficulties, ADLs, and IADLs among females in this study may be due to various reasons, such as the underdiagnosis of autism in females. That is, females may have to show worsened functional difficulties to meet diagnostic criteria for autism and therefore were included in the current sample. The increased incidence of functional difficulties and lessened flourishing among females in the current sample may also be attributed to camouflaging and possible under-recognized motor and social difficulties.

Federal poverty level of households influenced medical complications, ADLs, IADLs, behavior control, and school motivation. Children from high income households consistently showed better performance than those from low-income households, which provides evidence related to how social determinants of health influence function among autistic youth. Previous studies have established socioeconomic status is inversely related to prevalence of autism diagnoses, which is further stratified by race, with Black children consistently under diagnosed compared to White children [44,45]. The socioeconomic status, racial, and ethnic inequities in autism diagnoses and associated service provision are well established [46,47,48]. Gaps in service provision for marginalized children with autism result in fewer developmental supports, and children have fewer opportunities for engagement in various activities and therapies. For marginalized autistic children, caregivers have fewer access to services due to systemic barriers [49]. Such decreased supports and associated learning opportunities contribute to inequities in developmental outcomes, which is evidenced by factor scores in child function. Additionally, poverty influences health care access [50,51,52], which may account for the worsened medical complications evidenced by low-income groups in the current study. While the current study investigated child factors individually, the intersectionality of child factors may influence functional status. Intersectionality refers to the ways that different systems perpetuate inequities based on race, sex, gender, disability, and ethnicity [53]. While not considered in the current study, research may investigate how the intersectionality of child and family factors (e.g., race/ethnicity and sex) contribute to overall functioning status. The factors considered in this analysis (poverty, race/ethnicity) relate to social determinants of health and must be further researched in the context of systemic inequities that contribute to developmental disparities among autistic youth.

The current study was limited in that the NSCH relies on parent/caregiver self-report and may not align with behavioral or objective assessments of child function. Additionally, the NSCH is not representative of the United States population is likely not representative of the autistic population. Given the cross-sectional nature of NSCH data, causal relationships between factors associated with child function cannot be established by the current study. Lastly, findings are limited to the sample’s ages of 10–17 year olds and may not apply to younger children or autistic adults. Future research may investigate how the current study’s factor structure differs among children without autism. Further, future studies should replicate the factor structure found in the current analysis and factors related to flourishing and functional difficulties may be used in future studies to investigate the possibility of subtypes of autistic children to parse heterogeneity in function.

## Figures and Tables

**Table 1:** Participant Characteristics.

Variable	Participants Total n = 2959 n (%)
Sex	
Male	2360 (79.8)
Female	599 (20.2)
Autism Severity	
Mild	1539 (52)
Moderate-Severe	1393 (47.1)
Missing	27 (0.9)
Race/Ethnicity	
Asian	98 (3.3)
Black/African American	191 (6.5)
White	2135 (72.2)
Hispanic	316 (10.6)
Multi-racial, Other	212 (7.2)
Federal Poverty Level of Household *	
0–199%	1018 (34.4)
200–199%	458 (15.5)
300–399%	447 (15.1)
400%+	1036 (35.0)
Age (years)	Mean = 13.76 (SD = 2.24)
	Range = 10–17

* based on State Health Insurance Program.

**Table 2:** Model Fit Indices.

Statistic	Values
CFI	0.94
TLI	0.91
SRMR	0.05
RMSEA	0.08 (0.04–0.05), *p* = 0.93

**Table 3:** Confirmatory Factor Analysis Results.

Item	Factor Loading	SE	*p* Value
*Factor 1: Medical Difficulties*			
Difficulty eating or swallowing	0.824	0.049	<0.0001
Difficulty digesting food, including stomach/intestinal problems	0.691	0.041	<0.0001
Repeated or chronic physical pain, including headaches or other back or body pain	0.560	0.056	<0.0001
*Factor 2: IADLs*			
Serious difficulty concentrating, remembering, or making decisions	0.856	0.018	<0.0001
Difficulty doing errands alone, such as visiting a doctor’s office or shopping	0.847	0.018	<0.0001
*Factor 3: ADLs*			
Serious difficulty walking or climbing stairs	0.681	0.036	<0.0001
Difficulty dressing or bathing	0.974	0.032	<0.0001
*Factor 4: Social Competence*			
Is bullied, picked on, or excluded by other children ^R^	0.580	0.058	<0.0001
Bullies others, picks on them or excludes them ^R^	0.655	0.101	<0.0001
Has difficulty making and keeping friends ^R^	0.601	0.049	<0.0001
*Factor 5: Behavioral Control*			
Shows interest and curiosity in learning new things	0.622	0.016	<0.0001
Works to finish tasks he or she starts	0.741	0.013	<0.0001
Stays calm and in control when faced with a challenge	0.675	0.015	<0.0001
How well can you and this child share ideas or talk about things that really matter	0.560	0.017	<0.0001
*Factor 6: School Motivation*			
Does all required homework	0.776	0.013	<0.0001
Cares about doing well in school	0.808	0.013	<0.0001

^R^ Item was reverse scored.

**Table 4:** Inter-Factor Correlations.

	F1	F2	F3	F4	F5	F6
F1: Medical	1.0	0.562	0.566	−0.152	−0.287	−0.302
F2: ADLs		1.0	0.835	0.123	−0.701	−0.519
F3: IADLs			1.0	−0.190	−0.615	−0.504
F4: Social Competence				1.0	0.219	0.268
F5: Behavioral Control					1.0	0.819
F6: School Motivation						1.0

**Table 5:** Multivariate GLM Results.

	Sex	ASD Severity	Poverty	Race/Ethnicity
Medical	F(1,6) = 23.534 **	F(1,6) = 348.838 **	F(3,6) = 6.866 **	F(4,6) = 7.242 **
IADLs	F(1,6) = 11.068 **	F(1,6) = 818.652 **	F(3,6) = 6.671 **	F(4,6) = 1.303
ADLs	F(1,6) = 12.104 **	F(1,6) = 735.597 **	F(3,6) = 8.142 **	F(4,6) = 1.107
Social	F(1,6) = 0.668	F(1,6) = 68.842 **	F(3,6) = 1.869	F(4,6) = 11.913 **
Behavior	F(1,6) = 0.069	F(1,6) = 441.008 **	F(3,6) = 4.600 *	F(4,6) = 0.828
School	F(1,6) = 0.964	F(1,6) = 271.501 **	F(3,6) = 3.083 *	F(4,6) = 2.396 *

* *p* < 0.05. ** *p* < 0.01.

## Data Availability

Publicly available datasets were analyzed in this study. This data can be found here: https://www.childhealthdata.org/learn-about-the-nsch/NSCH, accessed on 4 March 2024.
